# Functional consequences of brain exposure to saturated fatty acids: From energy metabolism and insulin resistance to neuronal damage

**DOI:** 10.1002/edm2.386

**Published:** 2022-11-02

**Authors:** Karina Sánchez‐Alegría, Clorinda Arias

**Affiliations:** ^1^ Departamento de Medicina Genómica y Toxicología Ambiental Instituto de Investigaciones Biomédicas, Universidad Nacional Autónoma de México Ciudad de México Mexico

**Keywords:** energy metabolism, insulin resistance, mitochondrial dysfunction, neurodegeneration, palmitic acid, saturated fatty acids

## Abstract

**Introduction:**

Saturated fatty acids (FAs) are the main component of high‐fat diets (HFDs), and high consumption has been associated with the development of insulin resistance, endoplasmic reticulum stress and mitochondrial dysfunction in neuronal cells. In particular, the reduction in neuronal insulin signaling seems to underlie the development of cognitive impairments and has been considered a risk factor for Alzheimer's disease (AD).

**Methods:**

This review summarized and critically analyzed the research that has impacted the field of saturated FA metabolism in neurons.

**Results:**

We reviewed the mechanisms for free FA transport from the systemic circulation to the brain and how they impact neuronal metabolism. Finally, we focused on the molecular and the physiopathological consequences of brain exposure to the most abundant FA in the HFD, palmitic acid (PA).

**Conclusion:**

Understanding the mechanisms that lead to metabolic alterations in neurons induced by saturated FAs could help to develop several strategies for the prevention and treatment of cognitive impairment associated with insulin resistance, metabolic syndrome, or type II diabetes.

## Graphical abstract

Saturated fatty acids induce insulin resistance in neurons. Neuronal metabolism of palmitic acid alters mitochondrial function.

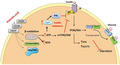

## 1 INTRODUCTION

Obesity is a growing health problem that has reached critical levels in recent decades. Epidemiologic studies from 2016 indicate that 13% of adults and 18% of children and adolescents under 19 years are obese worldwide,^1^ and it is estimated that obesity can affect 1.12 billion persons by 2030.^2^ This condition is primarily caused by nutritional imbalance associated with the high consumption of diets rich in fat and sugars and a sedentary lifestyle. Several studies have shown that HFD intake leads to elevated levels of saturated FAs in plasma and contributes to various health problems that reduce life expectancy and quality due to the development of chronic diseases such as insulin resistance, type II diabetes, metabolic syndrome and cardiovascular disease.^3^, ^4^, ^5^, ^6^, ^7^, ^8^ The HFD is characterized by a high content of saturated FA from animal fat, including myristate (C14:0), palmitate (C16:0), stearate (C18:0) and laurate (C12:0).^9^, ^10^, ^11^ Currently, it is recognized that high intake of saturated fat causes metabolic alterations not only in the peripheral organs, but also in the central nervous system (CNS). Although the mechanisms and signalling pathways affected in the brain by exposure to different concentrations of saturated FAs are not completely known, there is evidence of changes in energy metabolism,^12^, ^13^ reduced insulin sensitivity,^14^, ^15^, ^16^ increased ceramide production,^17^, ^18^ neuroinflammation^19^ and reduced neuronal viability.^20^, ^21^, ^22^, ^23^ Therefore, the chronic consumption of a HFD is considered a significant risk factor for cognitive decline, pathological brain aging and even AD.^24^, ^25^, ^26^, ^27^, ^28^, ^29^, ^30^, ^31^

Due to the complex nature of FA effects on the brain, in this review, we describe the current knowledge of the mechanisms involved in their transport from the circulation to the brain as well as the main metabolic routes activated in neurons, including evidence for neuronal β‐oxidation. Finally, we highlight the major mechanisms by which exposure to high levels of saturated FA can lead to neuronal dysfunction.

## 2 FATTY ACID UPTAKE IN THE BRAIN

Fatty acids are made up of a hydrocarbon chain with a terminal carboxyl group. They are classified as saturated when composed of single aliphatic chain bonds, monounsaturated with one double bond, and polyunsaturated with two or more double bonds. According to the chain length, FAs are divided into short‐chain FAs (2–4 carbon atoms), medium‐chain FAs (6–12 carbon atoms), and long‐chain FAs (14–18 carbon atoms).^32^, ^33^, ^34^ The physiological role of FAs depends on their length: short‐chain FAs are immediately available as an energy source, medium‐chain FAs can act as growth factors, and long‐chain FAs can act as structural components of cellular membranes and energy stores. The high levels of circulating long‐chain FA have been largely associated with atherogenic and thrombogenic diseases.^35^, ^36^, ^37^ In circulation, saturated FA concentrations are significantly increased in diabetic patients (350 μmol/L) compared with normal subjects (230 μmol/L).^38^ Additionally, PA is also increased in the cerebrospinal fluid in obese patients with poor cognitive performance.^39^

FAs travel in circulation mostly bound to albumin (95%), esterified into lipoproteins, and as unbound FAs (a very small proportion).^40^ Several studies have shown that FAs are able to cross the blood–brain barrier (BBB) and enter the brain to be taken up by endothelial cells, glial cells and neurons.^41^ Some experiments have shown that radio‐labelled FAs injected into the carotid artery of rats can be traced to neuronal cells,^42^ supporting the notion that plasma levels of FAs impact the type and concentration of lipid contents in the brain. How FAs cross the BBB and, in general, how they traverse the plasma membranes remains an open question that has generated two lines of evidence: one is that FAs can cross by simple diffusion, and the other proposes that they require a transport system mediated by specific proteins. It is recognized that both mechanisms can operate, but the protein‐dependent mechanism seems to be the prominent one.^43^ A number of FA transporters have been identified, including the FA transporter protein, which is composed of six isoforms with high homology between species (FATPs 1–6),^44^, ^45^, ^46^ and the FA translocase CD36, which has a high affinity for long‐chain FA transport and is also a receptor for lipoproteins.^47^, ^48^ The main proteins of the FATP family are members 1–4, which are located at the membranes of endothelial cells at the BBB and in grey matter. The transport through these proteins is dependent on chain length and the presence of double bonds.^46^, ^47^, ^49^ Once in the brain, short‐ and medium‐chain FAs enter the cell by a flip‐flop mechanism, while long‐chain FAs need FATPs to enter in a nonionized form. Similar to the BBB, neuronal FA uptake is specific and chain length‐dependent transport. Once FAs are transported to the cells, they bind to membrane or cytoplasmic FA binding proteins (FABPs) that help to organize them into specific intracellular domains for further utilization in different metabolic routes.^50^ For example, cytosolic fatty acid‐binding protein 3 (FABP3) is involved in arachidonic acid neuronal uptake but not in PA transport.^51^, ^52^, ^53^ CD36, now designated scavenger receptor B2,^54^, ^55^ has been found in endothelial, glial, and neuronal cells not only at the cell membrane, but also in intracellular compartments. It is thought that CD36 participates in FA dissociation from albumin, and it has been placed in the context of various mechanisms related to physiological and pathological lipid metabolism. In hypothalamic neurons, CD36 acts as part of a lipid‐sensing mechanism for the control of food intake^56^ and participates in the neurovascular dysfunction associated with AD.^57^

## 3 ARE NEURONS ABLE TO METABOLIZE FATTY ACIDS TO PRODUCE ENERGY?

It is known that 20% of the total energy requirement in the adult brain is obtained from the oxidation of FA.^58^ It was also long believed that astrocytes are the only brain cells able to metabolize FA and produce ^14^CO_2_ as an indicator of the β‐oxidation process.^59^, ^60^, ^61^, ^62^, ^63^, ^64^ However, it was reported that isolated neuronal mitochondria can oxidize palmitoyl carnitine in the presence of metabolic substrates.^65^ Neurons possess the necessary machinery for fatty acid β‐oxidation, and they also express long‐chain fatty acid acyl‐CoA synthetase, carnitine palmitoyltransferase Ia and c (CPT1a and CPT1c), and mitochondrial uncoupling protein 2 (UCP2). In fact, the CPT1c form is highly expressed in neurons.^66^, ^67^ Although the function of CPT1c is not yet clear, metabolomic analysis has revealed that it could play an alternative role in neuronal oxidative metabolism.^68^, ^69^, ^70^, ^71^, ^72^ Interestingly, some studies have shown that neuronal deficiency of this enzyme is associated with neurodegenerative diseases.^68^, ^69^, ^70^, ^71^, ^72^ Currently, it is proposed that neurons present low levels of β‐oxidation due to the limited activity of their mitochondrial enzymes. Comparing neurons with other cells of the periphery, the subsequent β‐oxidation enzymes have low activity. For example, the 3‐ketoacyl‐coenzyme A thiolase has only 0.7% activity, acyl‐CoA dehydrogenase 50%, and enoyl‐CoA‐dehydrogenase 19%. At the same time, the activity of the mitochondrial respiratory chain in neurons is lower than that in other brain cells, such as astrocytes. For this reason, ATP production from FAs is scarce in neurons, and when FAs must be oxidized in the mitochondria by a metabolic situation, this metabolic route produces high ROS levels, making neurons susceptible to oxidative damage.^73^, ^74^, ^75^ However, in some specific neurons, such as photoreceptors in the retina, FA oxidation seems to be the major source of energy to satisfy high metabolic demands.^76^ It was also reported in cultured rat cortical neurons that the inhibition of fatty acid synthase (FAS) leads to a decrease in the levels of ATP and activation of AMP‐dependent kinase (AMPK) as well as CPT1; such effects result in increased fatty acid oxidation to restore ATP levels sufficient to sustain neuronal activity and survival.^77^ FAS catalyses the condensation of acetyl‐CoA and malonyl‐CoA to generate long‐chain fatty acids and is highly expressed in neurons in different brain regions, including hypothalamic neurons that regulate feeding behaviour and systemic glucose levels.^78^ In a recent transcriptomic study, it was demonstrated that neurons can respond to high but not toxic concentrations of PA, increasing the expression of several genes involved in lipid and energy metabolism.^79^ Together, these data indicate that astrocytes are not only brain cells able to metabolize saturated FAs through β‐oxidation but also neurons can metabolize them under certain conditions.

Similar to other cells, neurons have another option to metabolize FAs. While short‐, medium‐ and long‐chain FAs are β‐oxidized in mitochondria, very‐long‐chain FAs (26:0) are metabolized in peroxisomes.^80^ The transport of FAs toward peroxisomes is mediated by the ABC transporter family through cycles of ATP binding and hydrolysis.^81^ There is also another mechanism of carnitine‐dependent transport of FAs in peroxisomes with less participation.^82^ FA oxidation in peroxisomes produces acetyl‐CoA or propionyl‐CoA, which are further metabolized in the mitochondria. Peroxisome‐mediated β‐oxidation has been proposed to be part of a homeostatic mechanism for hyperactive neurons to produce enough ATP to sustain neuronal functions. In summary, there is growing evidence that neurons can metabolize FAs by mitochondria or peroxisomes, depending on the chain length and energy requirements. In addition, if neurons increase their energy demands, neuron‐astrocyte metabolic coupling results in the best option to maintain energy homeostasis. Interestingly, neuron‐astrocyte coupling‐dependent FA detoxification has been also reported to prevent injury in the CNS. In cultured hippocampal neurons during excitotoxic stimulation with N‐methyl‐D‐aspartate (NMDA), saturated FAs are oxidized, and the resulting damaging oxidized FAs are released from neurons and taken up by astrocytes through a carrier protein. Then, these FAs are stored in lipid droplets and consumed for energy supply to neurons.^83^ However, the neuronal energy imbalance is sometimes not resolved by the astrocyte metabolic support, as has been recently demonstrated after CNS damage, wherein reactive astrocytes can release toxic FAs that may contribute to neuronal damage.^84^

## 4 ALTERATIONS IN NEURONAL METABOLISM ASSOCIATED WITH INTAKE OF SATURATED FAT

There is a strong correlation between chronic intake of HFD and the development of neuroinflammation and brain insulin resistance. The last effect was reported to be dependent on the increased phosphorylation of residue S307 of insulin receptor substrate‐2 in the hypothalamus^85^ and a reduction in the activation of residue Y608 of insulin receptor substrate‐1 in hippocampal neurons.^25^ According to positron emission tomography measurements using [^11^C]‐palmitate and [^18^F]‐fluoro‐6‐thia‐heptadecanoic acid in obese patients with metabolic syndrome, increased uptake and accumulation of FAs in different brain regions was found in comparison with healthy subjects.^86^ The brain accumulation of FAs can be explained by the obesity‐induced increase in the transporter FTP1, as was found in the prefrontal cortex in rats fed a HFD.^87^ Experiments in rats have shown that the increased levels of FAs in the brain result in metabolic changes consisting of lower brain glucose uptake and glucose transporters and alterations in glycolytic and acetate metabolism and central insulin resistance.^88^, ^89^ Insulin resistance could interfere with brain glucose utilization, resulting in a compensatory increase in saturated FA uptake and oxidation. The described metabolic abnormalities impact neuronal morphology and physiology, manifesting as decreased long‐term potentiation and reduced markers of synaptic plasticity.^25^, ^89^ To explain some of the brain alterations produced by the high intake of saturated fat, several mechanisms have been analysed and proposed as potential drivers of brain pathology. Among them, mitochondrial dysfunction, neuroinflammation and oxidative damage are prominent.^90^ In fact, HFD intake directly enhances ROS generation^91^ accompanied with elevated expression of NADPH oxidase enzyme.^92^ It was reported that consumption of a HFD reduces the levels of the mitochondrial fusion protein mitofusin 2 (MFN2) in hypothalamic neurons, resulting in loss of mitochondrial‐ER contacts and leading to ER stress and the development of leptin resistance.^93^ Mitochondrial‐ER contacts regulate mitochondrial shape and motility; thus, the loss of these contacts produce mitochondrial dysfunction and alters energy metabolism and the cellular redox state, inducing autophagy and inflammasome signalling.^94^, ^95^ These effects were corroborated in C57BL/6 mice fed a HFD, which presented a decrease in MFN2 expression in the arcuate nucleus.^96^ In addition, the mitochondrial dysfunction caused by the content of saturated FAs in the HFD also reduced the mitochondrial‐dependent Ca^2+^ uptake capacity that was accompanied by a decrease in hypothalamic neuronal excitability and consequent impaired function of energy control in the hypothalamus during obesity.^97^ Moreover, HFD alters the hypothalamus‐dependent regulation of body weight, changing the brain expression of diverse neuromodulators, such as neuropeptide Y (NPY), orexins and proopiomelanocortin (POMC).^98^, ^99^

## 5 PALMITIC ACID: THE MAIN SATURATED FATTY ACID UNDERLYING THE ADVERSE EFFECTS OF HFD IN NEURONS

PA is a 16‐carbon long‐chain saturated FA that is the most abundant saturated FA in the human body (65% of saturated FAs) and represents the main component of HFD. PA can be provided by the diet from vegetables (10%–40%) and animal fat (20–30%)^100^ or synthesized from amino acids, carbohydrates and other fatty acids in peripheral cells.^101^, ^102^, ^103^ An increase in circulating levels of PA has been considered the responsible factor for the development of several conditions, such as type II diabetes, cardiovascular diseases, pro‐metastatic activity and cognitive decline.^38^, ^102^, ^104^, ^105^, ^106^ In the brain, PA follows different metabolic routes, some of which could be associated with neuronal dysfunction. Among these, ceramide synthesis has been proven to have a causal role in insulin resistance and neuroinflammation.

### 5.1 Ceramide synthesis and neurotoxicity

Ceramides are signalling molecules involved in neuronal development, neuronal death and cellular senescence. De novo ceramide synthesis is controlled by the availability of palmitoyl‐CoA, which activates the rate‐limiting enzyme serine‐palmitoyl transferase.^107^ Depending on chain length, different species of ceramides are produced and serve different roles in cellular homeostasis, with ceramide 16 (C16) being the most involved in apoptosis.^108^ Exposure to a high concentration of PA induced the intracellular accumulation of C16 accompanied by proinflammatory cytokine production in cultured neurons.^18^ Similarly, PA activates the enzyme serine‐palmitoyl transferase, which is involved in the accumulation of ceramides in astrocytes, enhancing the release of cytokines and activating a signalling cascade in neurons that upregulates the pro‐amyloid enzyme BACE‐1.^109^ In peripheral cells, C24 and C16 also participate in the development of insulin resistance through PP2A‐dependent dephosphorylation and inactivation of Akt.^110^ Thus, the production of ceramides can be a critical mechanism linking the development of neuroinflammation and insulin resistance under high PA concentrations in the CNS.

### 5.2 Neuronal insulin resistance

**FIGURE 1:**
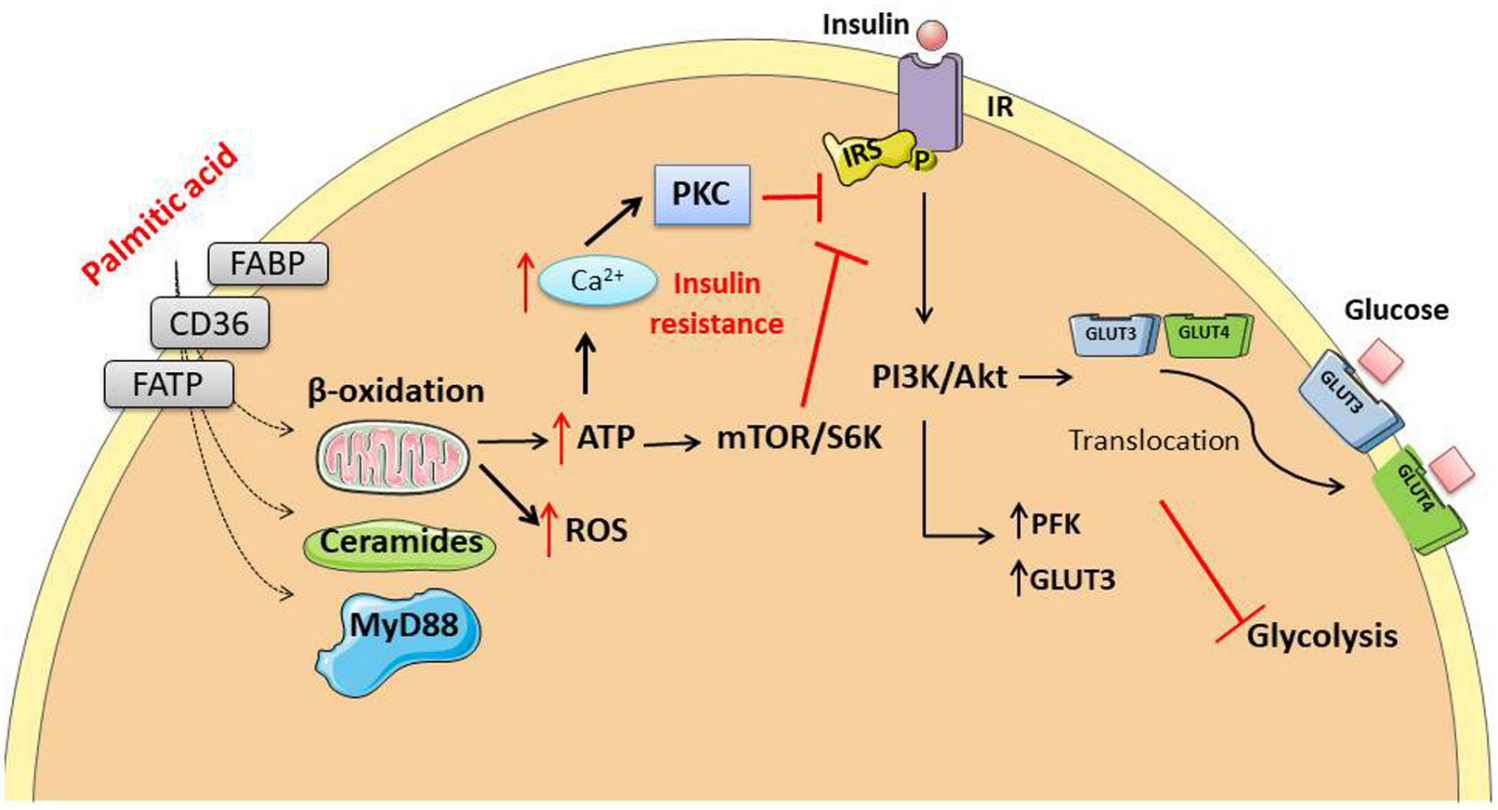
Palmitic acid causes insulin resistance by several mechanisms that include ROS and ceramide production as well as activation of MyD88 through PKCθ translocation to the cell membrane, which leads to internalization of the insulin receptor. Insulin resistance contributes to the impairment of glycolysis by decreasing the synthesis and activity of phospho‐fructose‐kinase and GLUT3 and impairing the translocation to the membrane of the glucose transporter, GLUT4.

The role of insulin in neuronal function as a metabolic, growth, synaptic and survival modulator has been extensively validated.^111^, ^112^, ^113^ Although neuronal glucose uptake is not dependent on insulin, the insulin/PI3K/Akt pathway regulates the expression of GLUT3 transporter and the glycolytic enzyme phosphofructokinase‐1 in neurons.^114^ After high neuronal firing rates, the activation of insulin signalling induces GLUT4 translocation to the membrane to increase glucose transport in hippocampal neurons. Thus, alterations in the insulin/PI3K/Akt pathway can also be associated with dysregulation of the energy balance and glucose homeostasis.^115^ Similarly, as it occurs in the peripheral organs, PA also contributes to the development of insulin resistance in neurons.^13^, ^16^, ^116^, ^117^, ^118^, ^119^ As previously stated, there is evidence that neurons can metabolize saturated FAs to produce energy, although it is still unknown under which conditions they can be used for this purpose. Recently, it has been reported for both, cultured rat cortical neurons and differentiated human neuroblastoma cells that there is a significant reduction in NAD^+^ contents and an increase in ATP levels after exposure to high but not toxic doses of PA, suggesting that neurons can utilize PA as an energy substrate when exposed to high concentrations of saturated FAs.^13^, ^16^, ^120^ The reduction in NAD^+^ was correlated with the blunted effects of PA on insulin‐induced metabolic activation as well as with the inhibition of the insulin/PI3K/Akt pathway. The participation of ROS production in this effect was demonstrated by the inhibition of mitochondrial ROS with the mitochondria‐targeted antioxidant mitoTEMPO, which prevented PA‐dependent insulin resistance.^16^ PA‐dependent insulin resistance was also associated with the translocation of protein kinase C‐θ (PKC‐θ) toward the cell membrane, which leads to the phosphorylation of the insulin receptor in inhibitory residues in hypothalamic neurons.^14^ Another mechanism involved in PA‐induced insulin resistance in neurons is the activation of MyD88, an essential signalling adaptor for most toll‐like receptors (TLRs) and members of the interleukin‐1 (IL‐1) receptor family. The activation of MyD88 by PA leads to a TLR‐4‐dependent inflammatory response that induces not only insulin resistance but also leptin resistance, which exacerbates the energy imbalance in neurons.^121^, ^122^, ^123^ The PA‐induced loss of insulin sensitivity seems to also be associated with PA‐dependent ATP production that mediates the opening of voltage‐dependent Ca^2+^ channels. The resulting increase in intraneuronal Ca^2+^ concentrations activates Ca^2+^‐dependent cPKC, which phosphorylates Akt in inhibitory residues, leading to its inactivation.^13^ The loss of insulin sensitivity induced by PA may explain the strong association between the intake of saturated fat and the increased risk of neuronal dysfunction (Figure 1).

### 5.3 Impairment of mitochondrial function in neurons

**FIGURE 2:**
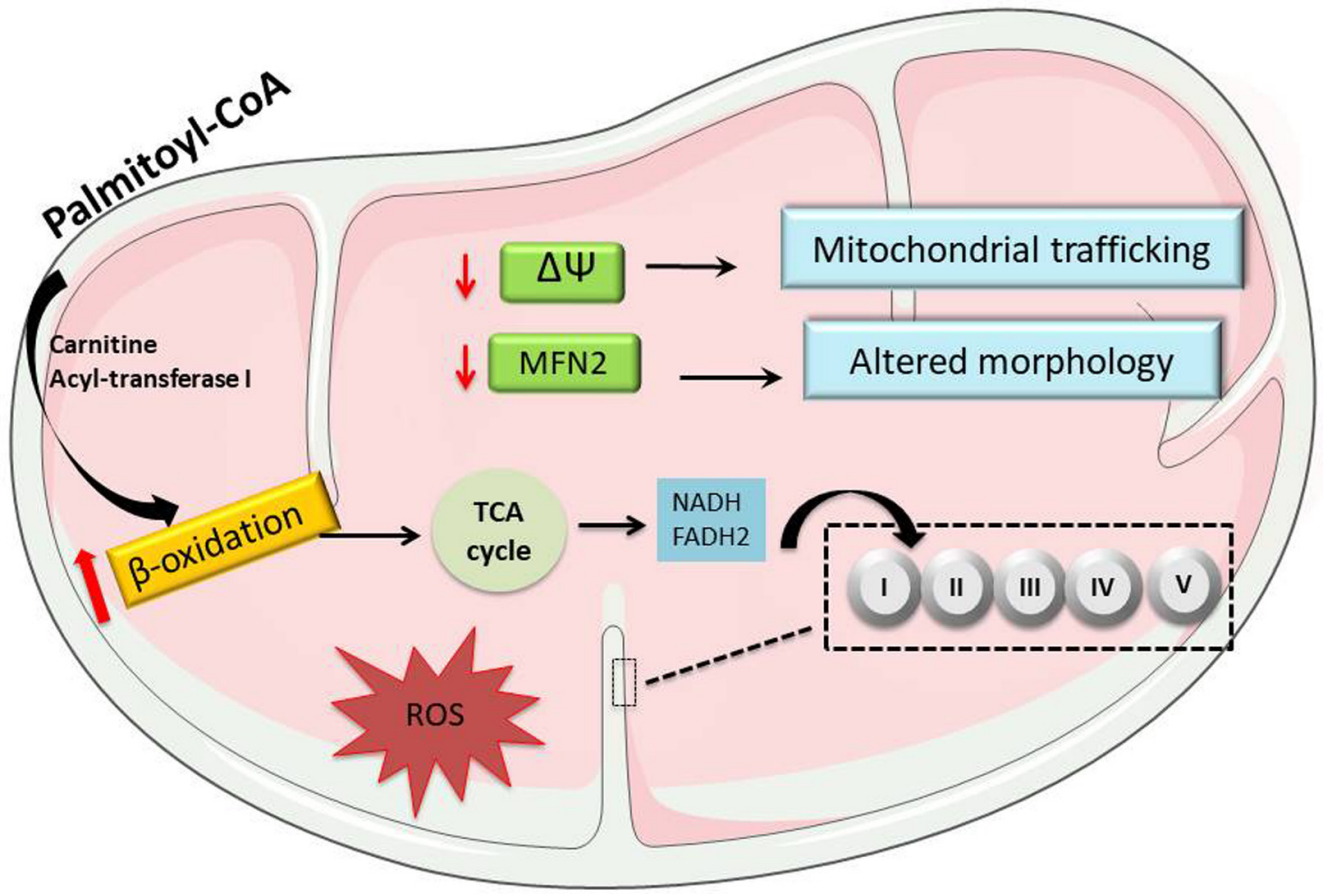
The possible route for β‐oxidation of palmitic acid in neurons is associated with the reduction of the mitochondrial fusion protein MFN2, decreased membrane potential, and ATP production as well as an increment in ROS levels. These conditions lead to decreased mitochondrial dynamics and bioenergetic capacity.

In addition to the above effects, PA exerts damaging consequences through enhances mitochondrial oxidative stress. PA treatment increased the production of superoxide dismutase after 8 h of exposure and decreased the production of ATP at 24 h, suggesting mitochondrial damage that correlates with the development of insulin resistance in neuroblastoma Neuro‐2a cells.^15^ A similar effect was observed in differentiated human neuroblastoma cells, in which treatment with PA for 1 h generated sustained ROS production that inhibited insulin signalling and insulin‐dependent mitochondrial activation.^16^ On the contrary, the exposure of hypothalamic cells to PA decreased the levels of the mitochondrial protein MFN2, ER stress and insulin resistance.^96^ Furthermore, in dorsal root ganglion (DRG) neuronal cultures exposed to PA for 24 h, a marked reduction in mitochondrial membrane potential and changes in ATP production were observed and accompanied by altered mitochondrial morphology and impaired mitochondrial trafficking.^124^, ^125^, ^126^ Overall, PA exposure affects neuronal metabolism, impairs mitochondrial function by loss of membrane potential and induces changes in mitochondrial morphology that result in inhibition of mitochondrial dynamics (Figure 2).

How might lipid‐overloaded mitochondria induce mitochondrial stress and insulin resistance? Although this question has not been fully resolved, it has been proposed that chronic elevation of fatty acids leads to persistent pressure on the electron transport chain, resulting in disruption of redox balance and ROS‐signalling.^127^ Since most studies have been conducted in peripheral cells, further research is necessary to understand the connection between mitochondrial overload, redox imbalance and insulin resistance in neurons chronically exposed to saturated fatty acids.

### 5.4 Endoplasmic reticulum stress

It has been demonstrated that HFDs trigger excessive endoplasmic reticulum (ER) stress and exert opposite influences on the expression of plasticity‐related proteins such as BDNF, synaptophysin and NMDA receptors in the rat prefrontal cortex.^87^ It is possible that many deleterious effects of HFD are exerted through its PA content, in view of the fact that in vivo and in vitro models have demonstrated that PA elicits the unfolded protein response and ER stress, is involved in the downregulation of leptin and insulin‐like growth factor receptor 1 expression in neurons^128^ and activates autophagy and apoptotic pathways. Accordingly, in cultured hypothalamic neurons, lipotoxicity induced by high doses of PA (0.7–1 mM) was accompanied by the activation of the ER stress pathway leading to the phosphorylation of the initiator of translation, eIF2α, and activation of the cleaved enzyme caspase‐3, an apoptotic effector molecule.^114^ Interestingly, some of the toxic effects of PA are prevented by the coadministration of the monounsaturated FA oleate.^15^ The protective role of oleate against PA‐induced apoptosis is probably mediated by conducting PA to incorporate into triglycerides and in this way to form a storage pool of lipid droplets, as shown in CHO cells and embryonic fibroblasts.^129^

## 6 ROLE OF GPR40 SIGNALLING ACTIVATION BY FATTY ACIDS

In addition to the consequences of the metabolic oxidation of PA on neurons, other effects can also be carried out through the activation of free fatty acid metabotropic receptors (FFARs). FFARs are G‐protein coupled receptors (GPCRs) located in the cell membrane that are mainly involved in metabolic regulation. FFARs are a family of four members: FFAR2 (also called GPR43) and FFAR3 (GPR41) are activated by short‐chain FFAs, whereas FFAR1 (GPR40) and FFAR4 (GPR120) are activated by medium‐ and long‐chain FFAs.^130^, ^131^, ^132^, ^133^, ^134^ Interestingly, GPR40 is highly expressed in neurons and is activated by docosahexaenoic acid (DHA) and selective agonists, but few studies have shown that PA activates these receptors in neurons.^13^, ^135^, ^136^

Activation of GPR40 regulates insulin secretion in pancreatic β‐cells, but its role in the CNS is not yet clear.^137^, ^138^, ^139^, ^140^, ^141^ It has been found that GPR40 activation by DHA protects against the adverse effects of neuroinflammation and insulin resistance in the brain.^142^ Additionally, signalling through GPR40 was found to be decreased in mice fed a HFD that developed cognitive deficits, but when GPR40 was activated by DHA or by its synthetic agonist, GW9508, improvements in cognitive functions resulted.^143^ Conversely, in hypothalamic neurons, the activation of GPR40 by PA contributes to the development of insulin resistance.^136^ In one study, in a human neuroblastoma model (SK‐N‐MC), it was found that PA‐mediated GPR40 signalling increased the expression of amyloid precursor protein (APP) and the catalytic enzyme BACE1, producing Aβ peptide through mTOR/p70S6K1‐mediated HIF‐1α expression and NF‐κB activation.^144^

**FIGURE 3:**
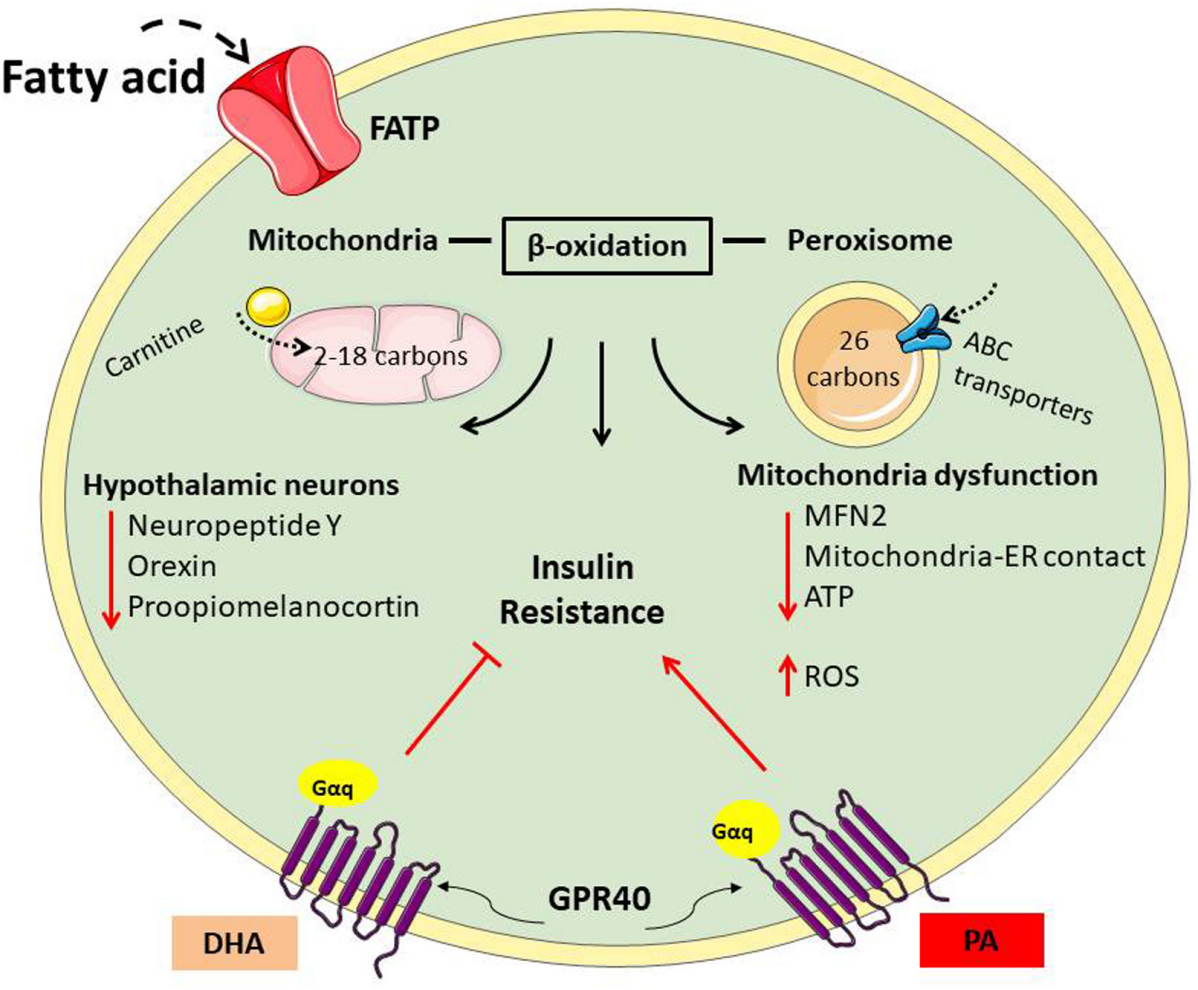
Fatty acids enter the brain and cells through FATPs. Within the cells, fatty acids (FAs) can be β‐oxidized by mitochondria (for FAs 2‐18 carbon atoms in length) or by peroxisomes (for 28‐carbon FAs). Palmitic acid metabolism can impact the hypothalamic energy sensing neurons, resulting in a loss of energy homeostasis. FAs can also activate GPR40 signalling associated with insulin resistance, but docosahexaenoic acid‐dependent activation of GPR40 improves insulin signalling.

In general, polyunsaturated FAs prevent and/or reverse the metabolic alterations induced by saturated FAs and manifest beneficial effects in neurodegenerative diseases.^145^, ^146^, ^147^, ^148^ Unlike PA, DHA is considered a positive modulator of the insulin pathway and promotes neuronal protection and survival.^149^ Due to its widely recognized influence on neuronal protection, DHA presents a compelling opportunity for the use of this nutritional therapy to counter the deleterious effects of saturated fat (Figure 3).

## 7 ASSOCIATION BETWEEN FATTY ACID METABOLISM AND ALZHEIMER'S DISEASE

Comorbidities associated with a high intake of saturated fat, such as obesity and type II diabetes, are risk factors for the development of cognitive impairments and even AD.^150^, ^151^, ^152^, ^153^, ^154^, ^155^

In fact, new evidence supports the relationship between obesity and dementia from a review of 19 longitudinal studies including people aged 35–65 years.^156^ AD is a neurodegenerative condition characterized by two hallmarks: the formation of amyloid plaques and the intraneuronal accumulation of neurofibrillary tangles by hyperphosphorylation of the cytoskeletal associated protein tau.^157^, ^158^, ^159^, ^160^, ^161^ Amyloid‐β protein (Aβ) originates from APP by the sequential enzymatic actions of β‐secretase and γ‐secretase.^162^, ^163^, ^164^, ^165^ The presence of high levels of saturated FAs and cholesterol in membrane lipid rafts was observed to contribute to the amyloidogenic processing of APP in APP/PSEN1 transgenic mice^166^, ^167^ and in astrocytes.^168^ Similarly, rats fed a HFD also showed increased APP and Aβ contents in the rat cerebral cortex,^169^ increased APP and BACE‐1 expression in the mouse brain^144^ and induced tau hyperphosphorylation in the rat hippocampus^25^ through the formation of an enzymatic complex that activates the enzyme GSK3β.^170^ Studies with 3xTg‐AD model have demonstrated that HFD treatment induces cognitive decline with or without increased Aβ levels.^171^, ^172^ In other AD model, the knock‐in mouse *App* ^NL−F/NL−F^, long‐term HFD intake was associated with insulin resistance, poor cognitive performance, increased deposition of Aβ and the presence of neuroinflammation and oxidative stress markers.^173^ Current research has shown an upregulation of BACE‐1 after PA exposure in cultured hippocampal neurons by a mechanism depending on the reduced activity of the deacetylase sirtuin 1^120^ and through PA‐mediated transcriptional activation of BACE‐1 in neurons after exposure to astrocyte‐conditioned media.^109^ Interestingly, a correlation between differential FA metabolism in specific brain areas and the development of some biochemical markers of AD has been shown. For example, cortical neurons exposed to conditioned medium obtained from PA‐treated cortical astrocytes expressed tau phosphorylation and BACE1, but not when neurons were exposed to cerebellar astrocyte media.^174^

These data demonstrate the effects of brain exposure to high levels of saturated FAs and point to the connection between lipid dyshomeostasis and the risk for AD.

## 8 CONCLUSIONS

The consumption of saturated FAs is strongly associated with morphological and functional changes in neurons. Accumulating evidence describes consistent dysregulation of neuronal metabolism induced by PA that leads to insulin resistance, decreased glycolysis, altered mitochondrial function and ER stress. These effects seem to contribute to cognitive decline. Recent interesting evidence supports the notion that in certain conditions, neurons can metabolize saturated long‐chain FAs through a metabolic energy pathway that sustains part of their deleterious effect. Understanding the mechanisms that lead to metabolic alterations in neurons could help to develop several strategies for the prevention, early detection and treatment of cognitive impairment associated with high consumption of saturated FAs or associated with insulin resistance, metabolic syndrome or diabetes. Although a variety of potential mechanisms have been explored, the underlying molecular cascade responsible for dietary fat‐induced neuronal dysfunction and behavioural changes remain elusive, and more research is needed to understand the signalling pathways that are activated in a specific metabolic context. An interesting avenue of studies to clarify the mechanisms downstream of the activation of GPR40 in neurons by polyunsaturated and saturated fatty acids is now open, as well as interrogation of the conditions that determine the metabolic routes that FAs follow into the brain to induce or avoid lipotoxicity.

## AUTHOR CONTRIBUTIONS

**Karina Sánchez‐Alegría:** Conceptualization (equal); data curation (equal); formal analysis (equal); funding acquisition (supporting); investigation (equal); methodology (equal); project administration (supporting); resources (supporting); software (equal); supervision (supporting); validation (supporting); visualization (supporting); writing – original draft (equal); writing – review and editing (supporting). **Clorinda Arias:** Conceptualization (equal); data curation (equal); formal analysis (equal); funding acquisition (lead); investigation (equal); methodology (equal); project administration (lead); resources (equal); software (equal); supervision (lead); validation (lead); visualization (lead); writing – original draft (equal); writing – review and editing (lead).

## CONFLICT OF INTEREST

The authors declare no conflict of interest.

## ETHICAL APPROVAL

The authors declare that neither animals nor human subjects were involved in the creation of this manuscript.

## Data Availability

Data sharing is not applicable to this article as no new data were created or analysed in this study.
